# Activity in nodose ganglia neurons after treatment with CP 55,940 and cholecystokinin

**DOI:** 10.14814/phy2.13927

**Published:** 2018-12-04

**Authors:** Juliane R. Johnston, Kimberly G. Freeman, Gaylen L. Edwards

**Affiliations:** ^1^ Department of Physiology and Pharmacology College of Veterinary Medicine The University of Georgia Athens Georgia

**Keywords:** Cannabinoid, food intake, vagus nerve, visceral afferent

## Abstract

Previous work has shown that cannabinoids increase feeding, while cholecystokinin (CCK) has an anorexigenic effect on food intake. Receptors for these hormones are located on cell bodies of vagal afferent nerves in the nodose ganglia. An interaction between CCK and endocannabinoid receptors has been suggested. The purpose of these studies is to explore the effect of pretreatment with a cannabinoid agonist, CP 55,940, on nodose neuron activation by CCK. To determine the effect of CP 55,940 and CCK on neuron activation, rats were anesthetized and nodose ganglia were excised. The neurons were dissociated and placed in culture on coverslips. The cells were treated with media; CP 55,940; CCK; CP 55,940 followed by CCK; or AM 251, a CB1 receptor antagonist, and CP 55,940 followed by CCK. Immunohistochemistry was performed to stain the cells for cFos as a measure of cell activation. Neurons were identified using neurofilament immunoreactivity. The neurons on each slip were counted using fluorescence imaging, and the number of neurons that were cFos positive was counted in order to calculate the percentage of activated neurons per coverslip. Pretreatment with CP 55,940 decreased the percentage of neurons expressing cFos‐immunoreactivity in response to CCK. This observation suggests that cannabinoids inhibit CCK activation of nodose ganglion neurons.

## Introduction

The vagus nerve has long been recognized as important in the control of thoracic and abdominal function (Grundy and Scratcherd 1989; Berthoud and Neuhuber 2000; Andrews and Sanger 2002; Hamilton and Raybould 2016). Vagal afferent nerve fibers carry information from cardiorespiratory receptors in the thorax and pressure sensors and chemical stimuli in the gastrointestinal tract to the dorsal medulla in order to aid in the control of cardiac, respiratory, and gastrointestinal function (Harper et al. 1959; Cummings and Overduin 2007; Andresen and Peters 2008). Specifically, activation of the vagus nerve slows gastric emptying via the vago‐vagal reflex in which afferent fibers send sensory information to the nucleus of the solitary tract (NTS) which communicates with the dorsal motor nucleus of the vagus (DMN) (Harper et al. 1959; Rogers et al. 1999; Travagli et al. 2006) The DMN then sends motor information through efferent fibers to stimulate or inhibit smooth muscle of the gastrointestinal tract (Rogers et al. 1999; Travagli et al. 2006). More recent work has suggested that the vagus nerve is important in sensing changes in behavior induced by altered gut microbiota (de Lartigue et al. 2011; Hamilton and Raybould 2016; Cawthon and de La Serre 2018).

Cholecystokinin (CCK) is a peptide that is involved in the control of food intake and slows gastric emptying by a vagally mediated mechanism (Gibbs et al. 1973a,b; Rehfeld 1981, 2004; Li and Owyang 1993; Reidelberger 1994; Noble and Rogues 1999; Valassi et al. 2008). This peptide binds to two different receptors, CCK‐1 and CCK‐2. CCK‐1 receptor (CCK‐1R) is located mostly in the gastrointestinal tract, and it is found on the gastric, celiac, and hepatic branches of the vagus nerve that communicate with the stomach, jejunum, and the hindbrain (Rehfeld 2004). Administration of CCK increases vagal afferent firing in the hindbrain region by action through binding to the CCK‐1R (Li and Owyang 1993; Cummings and Overduin 2007). The CCK‐2R is primarily found in the brain, notably in the hypothalamus and the hippocampus (Noble and Rogues 1999). CCK is released in response to food intake and activates vagal afferent fibers to decrease food intake and delay gastric emptying (Gibbs et al. 1973a; Smith and Gibbs 1975; Rehfeld 1981).

In addition to CCK, the endocannabinoid system is involved in the control of gastric motility and food intake, though whether or not this is vagally mediated is unclear (Gibbs et al. 1973b; Andrews and Sanger 2002). Endocannabinoids, such as anandamide and 2‐arachidonoyl glycerol, are lipophilic molecules formed from membrane glycerolphospholipids, and they are synthesized and secreted to physiological demand (Devane et al. 1988, 1992; Mechoulam et al. 1995; Sugiura et al. 2002; Izzo and Coutts 2005). These molecules bind to the CB_1_ and CB_2_ receptors (Devane et al. 1988, 1992; Mechoulam et al. 1995). The CB_1_ receptor has been found on neurons of the myenteric and submucosal plexuses of the gut, dorsal root ganglia, dorsal horn of the spinal cord, brainstem, and vagal efferent neurons (Herkenham et al. 1991a,b,c; Pertwee 2001). Within the medulla, abundant receptors are located in the NTS and the area postrema (AP), two areas important to visceral sensation (Matsuda et al. 1993; Tsou et al. 1998). The CB_2_ receptor is expressed in the brain, microglia, spleen, leukocytes, and the testes, and it has also been found in the lamina propria and submucosal plexus of the gut (Munro et al. 1993; Carlisle et al. 2002; Liu et al. 2009; Galiazzo et al. 2018). Both receptors are present in the nodose ganglia and nerve fibers involved in the vago‐vagal reflex (Rohof et al. 2012). Cannabinoid binding to CB_1_ receptors results in increased food intake and delayed gastric emptying, while binding to both CB_1_ and CB_2_ receptors results in analgesia and anti‐inflammatory effects in the gut (Hornby and Prouty 2004; Massa et al. 2004; Engel et al. 2010).

Many vagal afferent neurons, specifically those that originate from the stomach and duodenum, are located around the periphery of the nodose ganglion and contain both CB receptors and CCK‐1R (Burdyga et al. 2004, 2010; Rohof et al. 2012; Cluny et al. 2013). Studies have found that activation of CB_1_ receptors increases with fasting and that animals on high fat diets, or that are obese, have diminished changes in CB_1_ receptor activation to fasting and diet change (Burdyga et al. 2004, 2010; Cluny et al. 2013). Another study has found that costimulation of both CB receptor types is involved in the antiemetic actions of cannabinoid treatment in ferrets (van Sickle et al. 2005). Therefore, both CB receptors may be involved in the gastrointestinal effects associated with the endocannabinoid system. Additionally, CCK and cannabinoids have antagonistic effects on food intake through action on vagal afferent fibers (Burdyga et al. 2004; Orio et al. 2011). Based on these observations, we hypothesized that treatment with CP 55,940, a synthetic cannabinoid agonist that binds to both CB_1_ and CB_2_ receptors, will decrease activation of nodose ganglion neurons following treatment with CCK.

## Methods

Adult male Sprague‐Dawley rats (200–350 g) from Harlan Laboratories, Indianapolis, IN were used in all studies. The animals were housed in pairs in standard shoebox cages at 22°C with a 12:12 light:dark cycle and given access to food and water ad libitum. All research using animals was approved by the University of Georgia Institutional Animal Care and Use Committee.

### Extraction of nodose ganglia

Non‐fasted rats were anesthetized with a cocktail of ketamine (50 mg/kg of body weight), acepromazine (3.3 mg/kg of body weight), and xylazine (3.3 mg/kg of body weight). The rat was positioned in dorsal recumbency and an approximately 4 cm incision was made in the ventral neck over the trachea. The vagus nerve was identified using blunt dissection techniques to separate the digastric, sternocleidomastoid, and sternothyroid muscles to locate the nerve coursing beside the carotid artery. The nodose ganglia were identified near the posterior lacerated foramen and extracted. Once removed, the nodose ganglia were placed in Hibernate solution (Brain Bits; Springfield, IL) on ice until they were dispersed for cell culture.

### Cell isolation and culture

Primary cells were isolated and cultured according to the procedure outlined by Simasko et al. (2002). Both the left and the right nodose ganglia from an animal were combined for each cell isolation procedure. The ganglia were transferred along with 2–3 mL of Hibernate solution (Brain Bits Springfield, IL) into a 35 mm culture dish. The ganglia were de‐sheathed under a dissecting scope using sterile forceps and a 23‐gauge needle. Then, they were transferred into enzyme digestion solution (3 mg dispase, 3 mg collagenase, 3 mL Hank's Balanced Salt Solution without Calcium and Magnesium). The ganglia were minced using a scalpel and forceps. The dish was placed in an incubator (37°C, 5% CO_2_) to allow digestion for 90 min. Once the dish was removed, approximately half of the solution from the dish was pipetted into a 15 mL conical centrifuge tube using a pipette coated with Sigmacote (Sigma‐Aldrich, Saint Louis, MO). Then, using the same pipette, the ganglia were triturated in the dish. Following that, the ganglia were transferred into the centrifuge tube along with the rest of the solution in the culture dish. With a plain pipette, the culture dish previously containing enzyme digestion solution was filled with media made up of HEPES‐buffered DMEM supplemented with antibiotic (penicillin and streptomycin) and 10% Fetal Bovine Serum as to rinse the dish of any cells left. This media was transferred to the conical tube containing the minced ganglia. This was repeated until there was 10 mL of media in the conical tube. Then, the tube was centrifuged at 140 G for 1.5 min with no brake on. Once the tube was removed from the centrifuge, the supernatant was removed until 1.0 mL was left in the bottom. With pipettes coated with Sigmacote, the pellet was resuspended and more media was added until the total amount in the tube was 10 mL. Centrifugation was repeated. Finally, with another pipette coated with Sigmacote, the supernatant was removed once again until 1.0 mL remained at the bottom. The pellet was resuspended, and the solution was divided between six coverslips that had been pretreated with 0.1 mg/mL of poly‐L‐lysine hydrobromide (Sigma‐Aldrich Saint Louis, MO). The cells were placed in the incubator for approximately 3 hours, and then enough media was added to cover the coverslip and the slips were returned to the incubator. The following morning, the old media was removed and new media was added to each of the dishes.

### Cell treatment

Receptor selectivity and affinity for the pharmacological compounds used in these studies is described in a review by Pertwee (2010). The concentrations of CP 55,940, a nonselective CB agonist (Pertwee 2010), and AM251, a somewhat selective CB_1_ antagonist (Pertwee 2010), were selected to ensure saturation of receptors present on the cells in culture.

The cells were incubated in HDMEM + 10% FBS for 1 week before they were treated. The coverslips were randomly assigned to treatment groups and not prescreened for number of cells on each coverslip. Treatments included: media (Control); 1 *μ*mol/L CP 55,940 (Tocris; Bristol, UK); 0.1 *μ*mol/L CCK (American Peptide Company; Sunnyvale, CA); 1.0 *μ*mol/L CCK; 3 *μ*mol/L CCK; 10 *μ*mol/L CCK; 1 *μ*mol/L CP 55,940 with 1 *μ*mol/L CCK; 1 *μ*mol/L CP 55,940 with 3 *μ*mol/L CCK; or 1 *μ*mol/L AM251 (Tocris; Bristol, UK) with 1 *μ*mol/L CP 55,940 and 1 *μ*mol/L CCK. When treated with just CP 55,940, the CP 55,940 was left on the cells for 15 minutes, and then it was replaced with media for 90 min. The CCK treatments were left on for 90 minutes. The CP 55,940/CCK studies were performed by treating the cells with CP 55,940 for 15 minutes, and then replacing the CP 55,940 with CCK for 90 min. The AM/CP 55,940/CCK studies were conducted by combining the AM251 and CP 55,940 and treating the cells with the combined drugs for 15 minutes, then replacing the solution with CCK for 90 min. After treatment, the cells were fixed by immersion in 4% paraformaldehyde.

### Immunohistochemistry

The coverslips were first incubated in 0.3% H_2_O_2_ for an hour. Then, they were blocked in 4% normal goat serum for 2 hours. Two different antibodies were used in this experiment. The first antibody was rabbit polyclonal anti‐cFos from Calbiochem (Darmstadt, Germany) (1:40,000), and the second antibody was mouse monoclonal neurofilament from Sigma‐Aldrich (Saint Louis, MO) (1:500). The cells were incubated in these antibodies together for approximately 72 h. Following incubation, they were washed three times for twenty minutes in phosphate buffered saline (PBS) with Triton. Then, the protocol was followed from the rabbit Vectastain ABC Kit (Vector Laboratories, Inc; Burlingame, CA), and diaminobenzidine (DAB) peroxidase substrate (Vector Laboratories, Inc; Burlingame, CA) was used as the chromagen for the cFos staining. After the DAB was developed, the cells were washed three times for 20 minutes in PBS with Triton, and the mouse anti‐goat AlexaFluor 488 or 546 from Molecular Probes (Invitrogen; Grand Island, NY) was used as a fluorescent antibody to visualize the neurons in culture. Elimination of primary and secondary antibodies from the protocol was used as controls to ensure staining was accurate.

### Cell counting

All cell counts were conducted by a person blinded to the treatment applied to the coverslip. The total number of neurons stained with fluorescent secondary antibody to neurofilament was counted using a Nikon UFX‐IIA microscope. Those neurons that were also stained for cFos were counted as well. The percentage of cFos positive neurons was calculated based on the number of neurons that had also been stained with cFos. These numbers were compared between the different treatment groups.

### Statistical analysis

A one‐way ANOVA test of the CCK data, and of the agonist and antagonist data was used to determine whether the means of the treatment groups are significantly different at a *P* < 0.05 level. When ANOVA indicated significant differences, a Tukey's Multiple Comparison test was utilized to determine which specific treatments were significantly different from one another. Planned comparisons are noted, specifically 1 *μ*mol/L CCK versus 1 *μ*mol/L CP 55,940 + 1 *μ*mol/L CCK; 3 *μ*mol/L CCK versus 1 *μ*mol/L CP 55,940 + 3 *μ*mol/L CCK; and 1 *μ*mol/L CP 55,940 + 1 *μ*mol/L CCK versus 1 *μ*mol/L AM 251 + 1 *μ*mol/L CP 55,940 + 1 *μ*mol/L CCK. A value of *P* < 0.05 was used to indicate statistically significant differences. Data shown are mean ± SEM.

## Results

### Activation of nodose ganglia neurons with CCK

**Figure 1:**
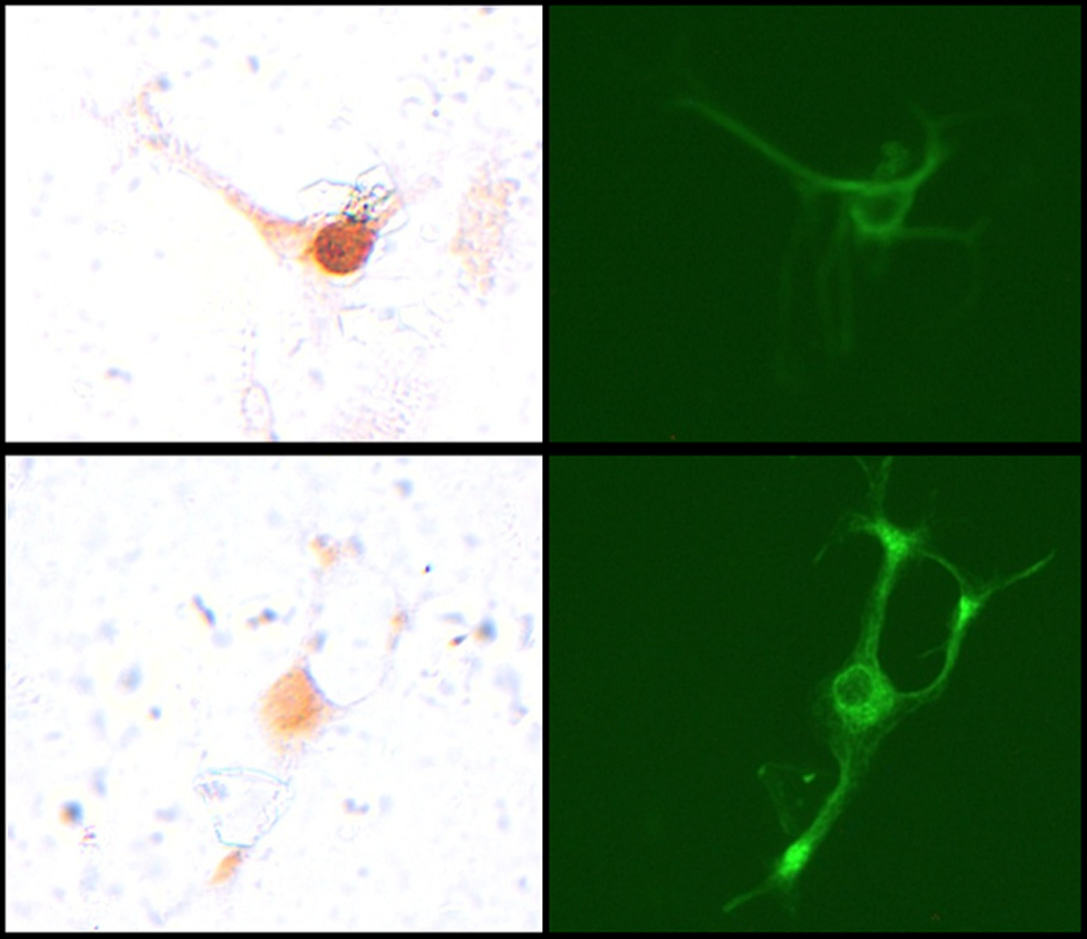
Photomicrographs of neurons stained for cFos (left panels) or neurofilament (right panels). The top panels illustrate a neuron staining positive for cFos. The bottom panels illustrate a neuron that is negative for cFos.

Examples of cells that were stained with neurofilament or cFos are illustrated in Figure 1. On average, there were 153 ± 13.0 neurons per coverslip (*n* = 113) that stained positive for neurofilament as shown by immunofluorescence.

**Figure 2:**
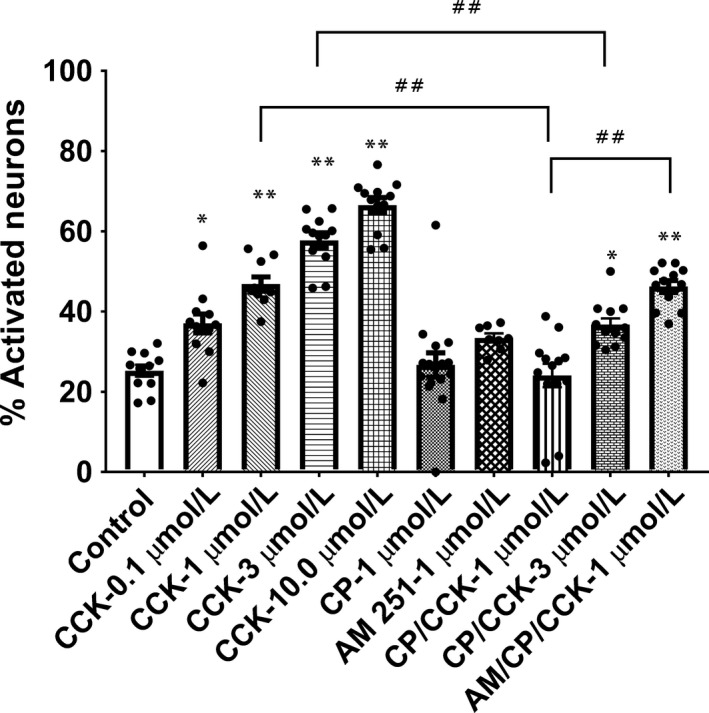
Percentage of neurons activated by the following treatments: media (Control); 0.1 *μ*mol/L CCK (CCK‐0.1 *μ*mol/L); 1.0 *μ*mol/L CCK (CCK‐1 *μ*mol/L); 3 *μ*mol/L CCK (CCK‐3 *μ*mol/L); 10 *μ*mol/L CCK (CCK‐10.0 *μ*mol/L); 1 *μ*mol/L CP 55,940 (CP‐1 *μ*mol/L); 1 *μ*mol/L AM251 (AM251‐1 *μ*mol/L); 1 *μ*mol/L CP 55,940 with 1 *μ*mol/L CCK (CP/CCK‐1 *μ*mol/L); 1 *μ*mol/L CP 55,940 with 3 *μ*mol/L CCK (CP/CCK‐3 *μ*mol/L); or 1 *μ*mol/L AM251 with 1 *μ*mol/L CP 55,940 and 1 *μ*mol/L CCK (AM/CP/CCK‐1 *μ*mol/L). Data were analyzed by 1 way ANOVA and post hoc Tukey's multiple comparisons test. *Significantly different from control (*P* < 0.02); **Significantly different from control (*P* < 0.0001). Planned comparisons: 1 *μ*mol/L CCK versus 1 *μ*mol/L CP 55,940 + 1 *μ*mol/L CCK; 3 *μ*mol/L CCK versus 1 *μ*mol/L CP 55,940 + 3 *μ*mol/L CCK; 1 *μ*mol/L CP 55,940 + 1 *μ*mol/L CCK versus 1 *μ*mol/L AM 251 + 1 *μ*mol/L CP 55,940 + 1 *μ*mol/L CCK (^##^ *P* < 0.0001).

Single factor ANOVA followed by a post hoc Tukey's multiple comparisons test showed that treatment with CCK significantly elevated the percentage of neurons activated at all doses tested, 0.1 to 10 *μ*mol/L ((*F*(9, 108) = 41.78, *P* < 0.0001; Tukey's, *P* < 0.02)). Of these neurons, 25 ± 1.5% of the control neurons (*n* = 11 coverslips, 165 ± 28.1 neurons/coverslip, avg) stained positive for cFos, indicating that these neurons had been activated. Thirty‐five percent ±1.7% of the neurons that were treated with 0.1 *μ*mol/L CCK (*n* = 11 coverslips, 314 ± 72.7 neurons/coverslip, avg); 47 ± 1.8% of them treated with 1.0 *μ*mol/L CCK (*n* = 10 coverslips, 126 ± 32.7 neurons/coverslip, avg); 58 ± 1.9% treated with 3.0 *μ*mol/L CCK (*n* = 12 coverslips, 191 ± 34.1 neurons/coverslip, avg); and 67 ± 1.9% treated with 10.0 *μ*mol/L CCK (*n* = 12 coverslips, 159 ± 41.6 neurons/coverslip, avg) also stained positive for cFos (Fig. 2). These data suggest a relationship between the amount of activation of nodose ganglia neurons and the concentration of CCK that was used to activate them. Thus, indicating a concentration‐dependent effect on vagal afferent neuron activation.

### Impact of a cannabinoid agonist, CP 55,940, on CCK activation of nodose ganglion neurons

Coverslips treated with CP 55,940 (1 *μ*mol/L) for 15 minutes prior to being incubated with media for 90 minutes (*n* = 14) revealed that there were 44 ± 6.6 neurons on average that stained positive for neurofilament on these coverslips. Of these, 26 ± 1.2% also stained positive for cFos (Fig. 2). This number did not significantly differ from the percentage of neurons that stain positive for cFos under control conditions (*P* > 0.9, Table 1). Therefore, CP 55,940 by itself did not alter cFos activation compared to control.

**Table 1:** An analysis of variance (ANOVA) yielded significant differences among treatment groups, *F*(9, 108) = 41.78, *P* < 0.0001. All comparisons and Adjusted P Values are shown for the Tukey's multiple comparisons test

Comparison Group	Adjusted *P* Value	Comparison Group	Adjusted *P* Value
Control vs. CCK‐0.1 *μ*mol/L	0.0131	CCK‐1 *μ*mol/L vs. AM/CP/CCK‐1 *μ*mol/L	>0.9999
Control vs. CCK‐1 *μ*mol/L	<0.0001	CCK‐3 *μ*mol/L vs. CCK‐10.0 *μ*mol/L	0.1483
Control vs. CCK‐3 *μ*mol/L	<0.0001	CCK‐3 *μ*mol/L vs. CP‐1 *μ*mol/L	<0.0001
Control vs. CCK‐10.0 *μ*mol/L	<0.0001	CCK‐3 *μ*mol/L vs. AM251‐1 *μ*mol/L	<0.0001
Control vs. CP‐1 *μ*mol/L	>0.9999	CCK‐3 *μ*mol/L vs. CP/CCK‐1 *μ*mol/L	<0.0001
Control vs. AM251‐1 *μ*mol/L	0.4051	CCK‐3 *μ*mol/L vs. CP/CCK‐3 *μ*mol/L	<0.0001
Control vs. CP/CCK‐1 *μ*mol/L	>0.9999	CCK‐3 *μ*mol/L vs. AM/CP/CCK‐1 *μ*mol/L	0.0135
Control vs. CP/CCK‐3 *μ*mol/L	0.0182	CCK‐10.0 *μ*mol/L vs. CP‐1 *μ*mol/L	<0.0001
Control vs. AM/CP/CCK‐1 *μ*mol/L	<0.0001	CCK‐10.0 *μ*mol/L vs. AM251‐1 *μ*mol/L	<0.0001
CCK‐0.1 *μ*mol/L vs. CCK‐1 *μ*mol/L	0.0985	CCK‐10.0 *μ*M vs. CP/CCK‐1 *μ*M	<0.0001
CCK‐0.1 *μ*mol/L vs. CCK‐3 *μ*mol/L	<0.0001	CCK‐10.0 *μ*mol/L vs. CP/CCK‐3 *μ*mol/L	<0.0001
CCK‐0.1 *μ*mol/L vs. CCK‐10.0 *μ*mol/L	<0.0001	CCK‐10.0 *μ*mol/L vs. AM/CP/CCK‐1 *μ*mol/L	<0.0001
CCK‐0.1 *μ*mol/L vs. CP‐1 *μ*mol/L	0.0207	CP‐1 *μ*mol/L vs. AM251‐1 *μ*mol/L	0.5837
CCK‐0.1 *μ*mol/L vs. AM251‐1 *μ*mol/L	0.9896	CP‐1 *μ*mol/L vs. CP/CCK‐1 *μ*mol/L	0.996
CCK‐0.1 *μ*mol/L vs. CP/CCK‐1 *μ*mol/L	0.0021	CP‐1 *μ*mol/L vs. CP/CCK‐3 *μ*mol/L	0.0293
CCK‐0.1 *μ*mol/L vs. CP/CCK‐3 *μ*mol/L	>0.9999	CP‐1 *μ*mol/L vs. AM/CP/CCK‐1 *μ*mol/L	<0.0001
CCK‐0.1 *μ*mol/L vs. AM/CP/CCK‐1 *μ*mol/L	0.1132	AM251‐1 *μ*mol/L vs. CP/CCK‐1 *μ*mol/L	0.1859
CCK‐1 *μ*mol/L vs. CCK‐3 *μ*mol/L	0.0408	AM251‐1 *μ*mol/L vs. CP/CCK‐3 *μ*mol/L	0.9948
CCK‐1 *μ*mol/L vs. CCK‐10.0 *μ*mol/L	<0.0001	AM251‐1 *μ*mol/L vs. AM/CP/CCK‐1 *μ*mol/L	0.0146
CCK‐1 *μ*mol/L vs. CP‐1 *μ*mol/L	<0.0001	CP/CCK‐1 *μ*mol/L vs. CP/CCK‐3 *μ*mol/L	0.0031
CCK‐1 *μ*mol/L vs. AM251‐1 *μ*mol/L	0.013	CP/CCK‐1 *μ*mol/L vs. AM/CP/CCK‐1 *μ*mol/L	<0.0001
CCK‐1 *μ*mol/L vs. CP/CCK‐1 *μ*mol/L	<0.0001	CP/CCK‐3 *μ*mol/L vs. AM/CP/CCK‐1 *μ*mol/L	0.0867
CCK‐1 *μ*mol/L vs. CP/CCK‐3 *μ*mol/L	0.0759		

### Effect of pretreatment of neurons activated by CCK with CP 55,940

One‐way ANOVA of the cannabinoid agonist and antagonist data indicated that there were differences in the mean number of neurons activated with the various treatments ((*F* (9, 108) = 41.78)). Post hoc Tukey's multiple comparisons tests suggest that pretreatment with CP 55,940 has a significant effect on the percent of neurons activated by CCK at both the 1 *μ*mol/L and 3 *μ*mol/L concentrations (Fig. 2; *P* < 0.0001).

Nodose ganglia neurons treated with CP 55,940 (1 *μ*mol/L) for 15 minutes prior to treatment with 1 *μ*mol/L CCK for 90 min (*n* = 11 coverslips, 139 ± 17.4 neurons/coverslip, avg) revealed that approximately 28 ± 1.6% of those neurons showed cFos immunoreactivity compared to 47 ± 1.8% without pretreatment with CP 55,940 (Fig. 2). This demonstrated a significant decrease in neuron activation after pretreatment by CP 55,940 (*P* < 0.0001). Moreover, this percentage activation was not significantly different from control (*P* > 0.9, Table 1).

Examination of nodose ganglia neurons treated with CP 55,940 (1 *μ*mol/L) for 15 minutes prior to treatment with 3 *μ*mol/L CCK for 90 min revealed that 37 ± 1.6% of the neurons were activated (*n* = 12 coverslips, 80 ± 32.6 neurons/coverslip, avg) as evidenced by staining positive for cFos (Fig. 2). This was significantly different from the percentage activated with 3 *μ*mol/L CCK alone, 58 ± 1.9% (*P* < 0.0001). This percentage activated was also significantly different from control (*P* < 0.02, Table 1).

### Effect of the cannabinoid inverse agonist, AM251, on the reversal of activation with CCK by a cannabinoid agonist

We lastly evaluated whether an antagonist to the cannabinoid receptor could block the action of CP 55,940 on CCK‐activated nodose ganglion neurons. These coverslips were treated with a combination of 1 *μ*mol/L AM251 and 1 *μ*mol/L CP 55,940 for 15 minutes and then treated with 1 *μ*mol/L CCK for 90 min. Coverslips that were pretreated with AM251 in conjunction with CP 55,940 (*n* = 12 coverslips, 203 ± 27.3 neurons/coverslip, avg) revealed that 46 ± 1.5% of neurons stained positive for cFos (Fig. 2). This number was significantly different from control (*P* < 0.0001), but it was not significantly different from the percentage of neurons activated with 1 *μ*mol/L CCK (*P* > 0.9, Table 1). When coverslips were treated solely with AM251 as a control (*n* = 8 coverslips, 125 ± 26.7 neurons/coverslip, avg), the average percentage of neurons positive for cFos was 33 ± 1.1% (Fig. 2). This was not significantly different from the control (*P* > 0.4, Table 1), but was significantly different from the AM/CP 55,940/CCK treatment (*P* < 0.02, Table 1). These data suggest that in part by blocking the binding of CP 55,940 to cannabinoid receptors with AM251, the inhibition of neuronal activation was abolished. The affinity for AM251 for the CB_1_ receptor is greater than for the CB_2_ receptor (Pertwee 2010). Thus, this observation suggests that the decrease in activation of nodose ganglion neurons by CCK using CP 55,940 most likely involves the CB_1_ receptor. However, a role for the CB_2_ receptor cannot be ruled out.

## Discussion

Our working hypothesis is that an exogenous CB agonist acting via the CB_1_ receptor decreases activation of nodose ganglion neurons induced by treatment with CCK. Our results indicate that pretreatment with a CB receptor agonist, CP 55,940, causes a decrease in nodose neuron activation by CCK of neurons in culture.

Our data indicate that treatment of cultured vagal afferent neurons with CCK increases the number of neurons expressing cFos immunoreactivity. The number of cells activated increases with the increasing concentration of CCK applied to the neurons in culture and suggests a concentration dependence of this effect. Once we established the action of CCK to increase cFos immunoreactivity in nodose ganglion neurons, we were able to demonstrate that pretreatment with CP 55,940 decreased neuron activation occurring in response to 1 *μ*mol/L and 3 *μ*mol/L CCK. It is important to note that the dose of CP 55,940 we used was less effective against activation of neurons by 3 *μ*mol/L CCK than against activation by 1 *μ*mol/L CCK. This likely reflects the greater stimulation of the nodose ganglion neurons by the higher concentration of CCK as suggested by our concentration‐dependent increase in cFos immunoreactivity when treated with CCK. Finally we demonstrated that treatment of the nodose ganglion neurons with AM251 almost completely reversed the effect of CP 55,940 on 1 *μ*mol/L CCK activation of nodose ganglion neurons. This suggests that the decrease in activation to CCK by CP 55,940 is due to binding to the CB receptors, likely CB_1_ receptors.

It should be noted that the number of neurons on coverslips that stained positive for neurofilament was below the combined average in some of the treatment groups. Specifically, the coverslips treated with CP 55,940 alone and treated with CP 55,940/3 μmol/L CCK had fewer counted neurons. In order to avoid investigator bias, there was no effort made to normalize the coverslips in each group by cell density. The coverslips were randomly assigned to treatment groups. Thus, one cause for this difference is the variability of the concentration of cells that were plated, and survived in culture, on the coverslips that were randomly assigned to these treatment groups.

CCK is released by intestinal cells in response to food intake as a satiety signal to the brain (Valassi et al. 2008), while endocannabinoid release increases during the fasted state which correlates with the increased food intake that occurs subsequent to fasting (Pertwee 2001; Izzo and Sharkey 2010). CB_1_ receptor activation changes according to the animal's fed or fasted state, and there is an increase in CB_1_ activation during the fasted state (Burdyga et al. 2004; Cluny et al. 2013). However, a more recent report suggests that the strain of animals and type of food the animal is maintained on can influence the expression of cannabinoid receptors (Cluny et al. 2013). Orio et al. has also found that antagonizing the CB_1_ receptor and CCK administration have an additive effect on food intake suppression. Moreover, CB_1_ agonists counteract the effects of CCK on food intake when both are administered systemically, which indicates that there may be modulation of the CCK satietogenic system by the endocannabinoid system (Orio et al. 2011). Our study suggests that there may be a vagal component to this action.

It has been shown that activation of the CCK1R causes CREB phosphorylation through a PKC‐dependent pathway, which increases expression of CART and Y2R (de Lartigue et al. 2007). Our results suggest that endogenous cannabinoids can affect the phenotype of vagal afferent neurons. Cuellar and Isokawa have shown that 2‐AG inhibits ghrelin‐induced phosphorylation of CREB (Cuellar and Isokawa 2011). Therefore, cannabinoid binding to the CB_1_ receptor has been shown to decrease the amount of CREB phosphorylation in neuronal populations. It is possible that the decrease in neuronal activation by CP 55,940 in response to CCK is due to action involving the CREB pathway by affecting CREB phosphorylation. This phosphorylation can occur in response to calcium influx. In addition, CREB regulates cFos expression within the cell, which further supports the possibility that our results are due to action through this specific pathway. Future studies will evaluate the ability of CP 55,940 to impact CREB phosphorylation and calcium signaling.

Since the action of cannabinoids on behaviors such as food intake may be at multiple levels of the neuroaxis (Horn et al. 2018), it is difficult to ascribe the interaction of cannabinoids and CCK to any one site when pharmacological agonists and antagonists are administered systemically. Near‐arterial delivery of materials to the nodose ganglion is possible (Lacolley et al. 2006), but it would be very difficult to perform these studies in awake, behaving animals. Thus, directly testing the effects of nodose ganglion treatment with cannabinoid agonists and antagonists in whole animals on the satiating effects of CCK has not been conducted. Testing the effects of cannabinoid agonists and antagonists on gastrointestinal motility in anesthetized animals may be more tenable.

The physiologic role of cannabinoids in the vagal afferent system is still unclear, but they have historically been used in the treatment of gastrointestinal pain, flatulence, gastroenteritis, Crohn's disease, diarrhea, and diabetic gastroparesis, and are reported to cause an increase in appetite (Izzo et al. 2001). Recent studies have suggested a role for cannabinoids in the regulation of gastrointestinal function, food intake, and obesity in response to changes in gut microbiota (Muccioli et al. 2010; Cluny et al. 2015). Our data suggest that one role of cannabinoids in the visceral afferent system is to antagonize the actions of CCK. This could impact food intake and gastrointestinal motility. The potential for therapeutic benefit from cannabinoids is significant. There are multiple instances when increasing appetite is beneficial. The elderly, those undergoing chemotherapy, and the chronically ill all experience a decrease in appetite (Plata‐Salaman 1996; de Boer et al. 2013; Childs and Jatoi 2018), which leads to a decrease in energy intake. Energy is required for appropriate immune and inflammatory responses to fight infection and allow regeneration of damaged tissue. Identifying peripheral targets for cannabinoid action is important as these targets may provide a means of allowing beneficial effects of the cannabinoid while avoiding the central neural effects, such as dysphoria.

## Conclusions

We found that CCK increased the number of neurons expressing cFos in primary cultures of vagal afferent neurons in a concentration‐dependent fashion. The action of CCK on nodose ganglion neurons was antagonized by a cannabinoid agonist, CP 55,940. This action was reversed by the cannabinoid receptor antagonist AM 251. These findings are consistent with the hypothesis that cannabinoids act on vagal afferent fibers to antagonize satietogenic signals originating from the gut.

## Conflict of Interest

None declared.
